# The genome sequence of the slender grass hoverfly, *Melanostoma scalare *(Fabricius, 1794)

**DOI:** 10.12688/wellcomeopenres.20171.1

**Published:** 2023-10-23

**Authors:** William L.S. Hawkes, Karl R. Wotton, University of Oxford and Wytham Woods Genome Acquisition Lab, Darwin Tree of Life Barcoding collective, Wellcome Sanger Institute Tree of Life programme, Wellcome Sanger Institute Scientific Operations: DNA Pipelines collective, Tree of Life Core Informatics collective, Darwin Tree of Life Consortium

**Affiliations:** 1 Swiss Ornithological Institute, Sempech, Switzerland; 2 Centre for Ecology and Conservation, University of Exeter, Penryn, England, UK

**Keywords:** Melanostoma scalare, slender grass hoverfly, genome sequence, chromosomal, Diptera

## Abstract

We present a genome assembly from an individual male *Melanostoma scalare* (the slender grass hoverfly; Arthropoda; Insecta; Diptera; Syrphidae). The genome sequence is 738.2 megabases in span. Most of the assembly is scaffolded into 5 chromosomal pseudomolecules, including the X and Y sex chromosomes. The mitochondrial genome has also been assembled and is 16.08 kilobases in length.

## Species taxonomy

Eukaryota; Metazoa; Eumetazoa; Bilateria; Protostomia; Ecdysozoa; Panarthropoda; Arthropoda; Mandibulata; Pancrustacea; Hexapoda; Insecta; Dicondylia; Pterygota; Neoptera; Endopterygota; Diptera; Brachycera; Muscomorpha; Eremoneura; Cyclorrhapha; Aschiza; Syrphoidea; Syrphidae; Syrphinae; Melanostomini; *Melanostoma*; *Melanostoma scalare* (Fabricius, 1794) (NCBI:txid92598).

## Background

The slender grass hoverfly, *Melanostoma scalare*, is a medium to small hoverfly. This is one of the commonest in the UK, and along with other members of the genus, is one of the most abundant hoverflies of the Palaeartic ( Haarto & Ståhls, 2014). These hoverflies are black and yellow in colouration and are sexually dimorphic. Males of this species are narrowly built and elongate, making them rather distinctive ( Ball & Morris, 2015). Females have triangular abdominal markings and resemble female *Melanostoma mellinum* but are more elongate and have larger dust spots on their frons ( Stubbs & Falk, 2002).

*Melanostoma scalare* larvae are thought to be generalist predators on aphids and other small arthropods found in leaf litter or tussocky grass ( Wilkinson & Rotheray, 2017). While many other aphidophagous hoverfly lay only on previously infested plants, *M. scalare* females will lay on plants even in the absence of aphids, perhaps as because of their generalist food preferences ( Chandler, 1968). This behaviour lends the species to pest management options in agricultural settings ( Chandler, 1968). Adults have a long flight period spanning from March to November ( Stubbs & Falk, 2002). The adults feed on a wide variety of flowers making them important generalist pollinators ( Doyle *et al.*, 2020). The mitochondrial genome of this species has been previously sequenced ( Liu *et al.*, 2020). The production of a high quality *Melanostoma scalare* genome sequence described here, generated as part of the Darwin Tree of Life project, will further aid understanding of the biology and ecology of this hoverfly.

## Genome sequence report

**Figure 1.**
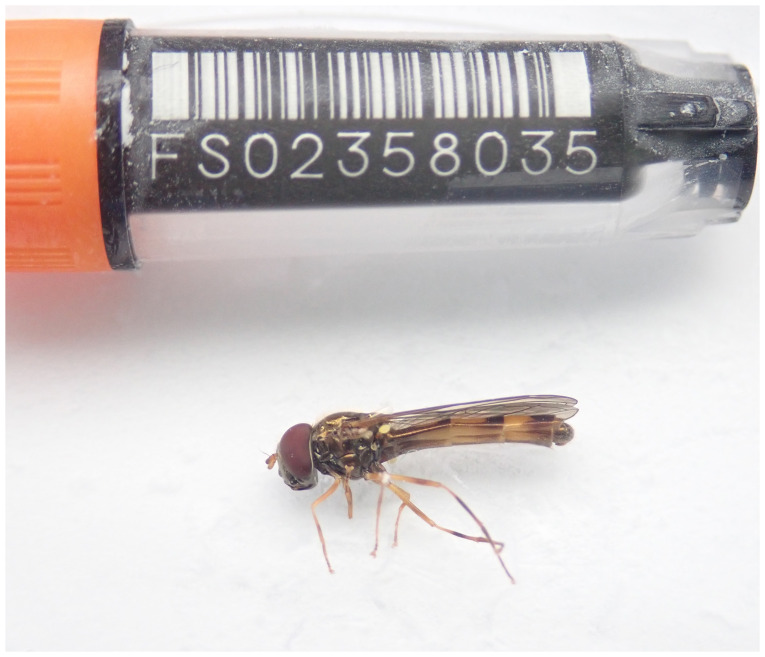
Photograph of the *Melanostoma scalare* (idMelScal2) specimen used for genome sequencing.

The genome was sequenced from one male *Melanostoma scalare* ( Figure 1) collected from Wytham Woods, Oxfordshire, UK (51.77, –1.34). A total of 31-fold coverage in Pacific Biosciences single-molecule HiFi long was generated. Primary assembly contigs were scaffolded with chromosome conformation Hi-C data. Manual assembly curation corrected 157 missing joins or mis-joins and removed 34 haplotypic duplications, reducing the assembly length by 0.84% and the scaffold number by 48.2%, and increasing the scaffold N50 by 120.8%.

**Figure 2.**
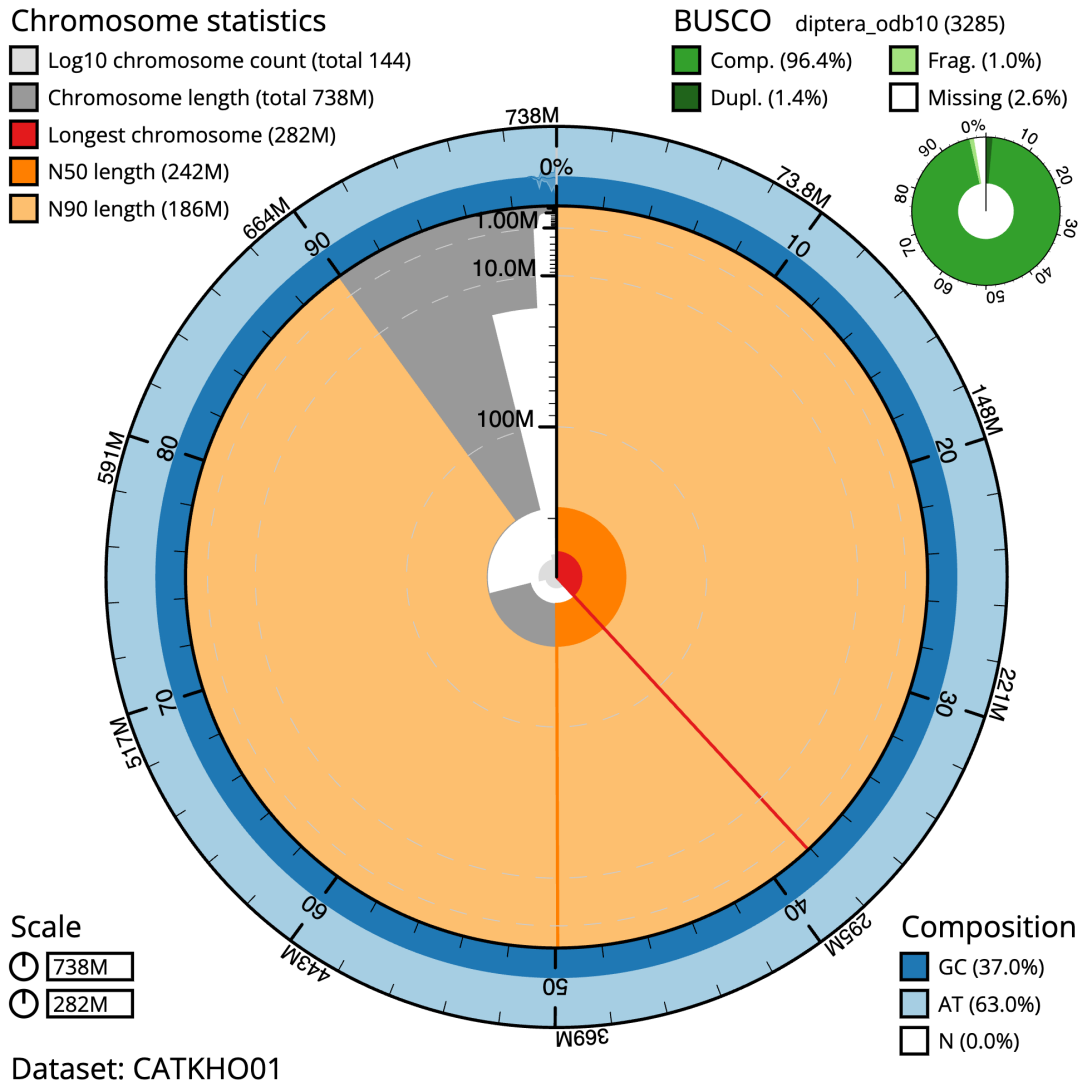
Genome assembly of *Melanostoma scalare*, idMelScal2.1: metrics. The BlobToolKit Snailplot shows N50 metrics and BUSCO gene completeness. The main plot is divided into 1,000 size-ordered bins around the circumference with each bin representing 0.1% of the 738,179,629 bp assembly. The distribution of scaffold lengths is shown in dark grey with the plot radius scaled to the longest scaffold present in the assembly (281,865,375 bp, shown in red). Orange and pale-orange arcs show the N50 and N90 scaffold lengths (241,717,587 and 185,741,843 bp), respectively. The pale grey spiral shows the cumulative scaffold count on a log scale with white scale lines showing successive orders of magnitude. The blue and pale-blue area around the outside of the plot shows the distribution of GC, AT and N percentages in the same bins as the inner plot. A summary of complete, fragmented, duplicated and missing BUSCO genes in the diptera_odb10 set is shown in the top right. An interactive version of this figure is available at https://blobtoolkit.genomehubs.org/view/Melanostoma%20scalare/dataset/CATKHO01/snail.

**Figure 3.**
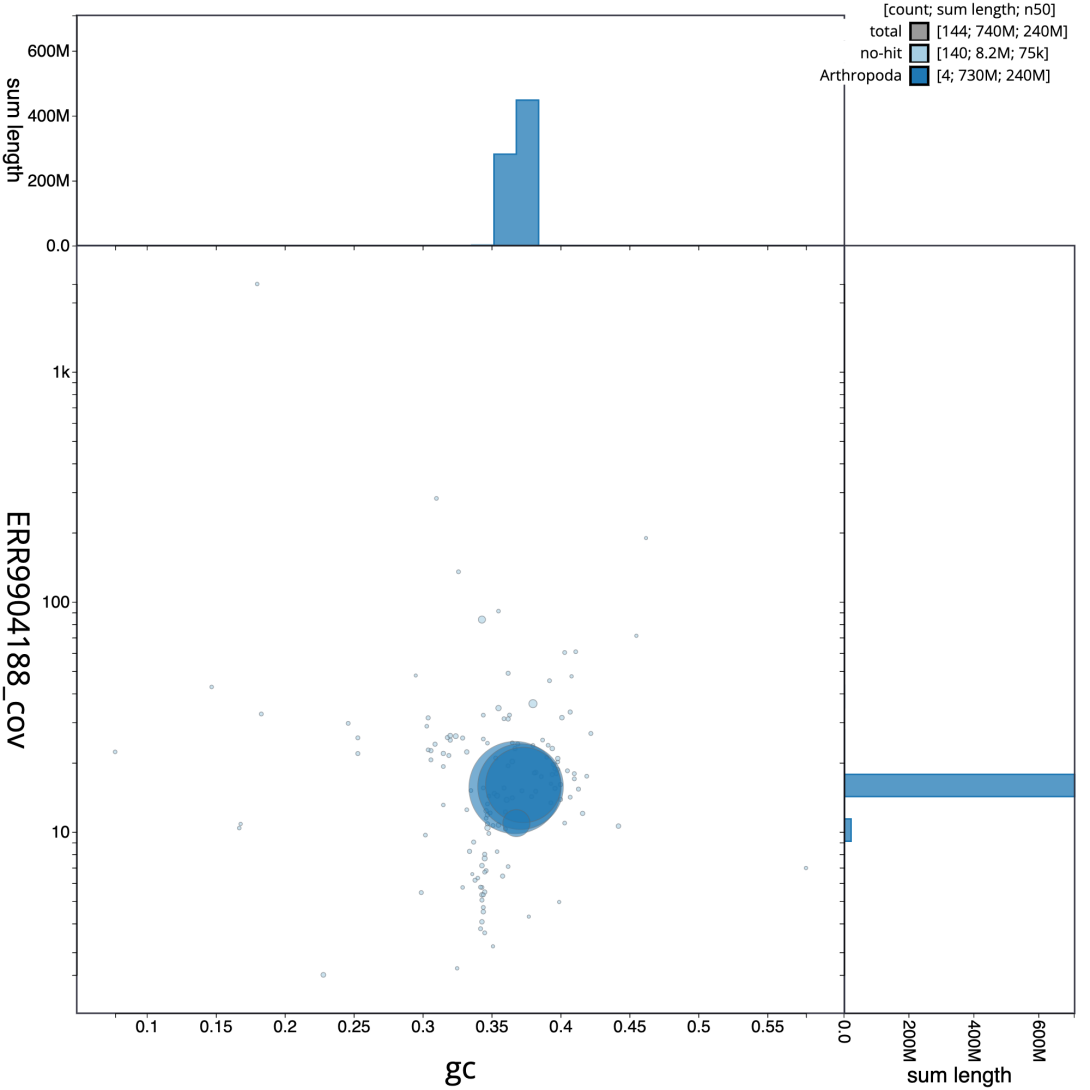
Genome assembly of *Melanostoma scalare*, idMelScal2.1: BlobToolKit GC-coverage plot. Scaffolds are coloured by phylum. Circles are sized in proportion to scaffold length. Histograms show the distribution of scaffold length sum along each axis. An interactive version of this figure is available at https://blobtoolkit.genomehubs.org/view/Melanostoma%20scalare/dataset/CATKHO01/blob.

**Figure 4.**
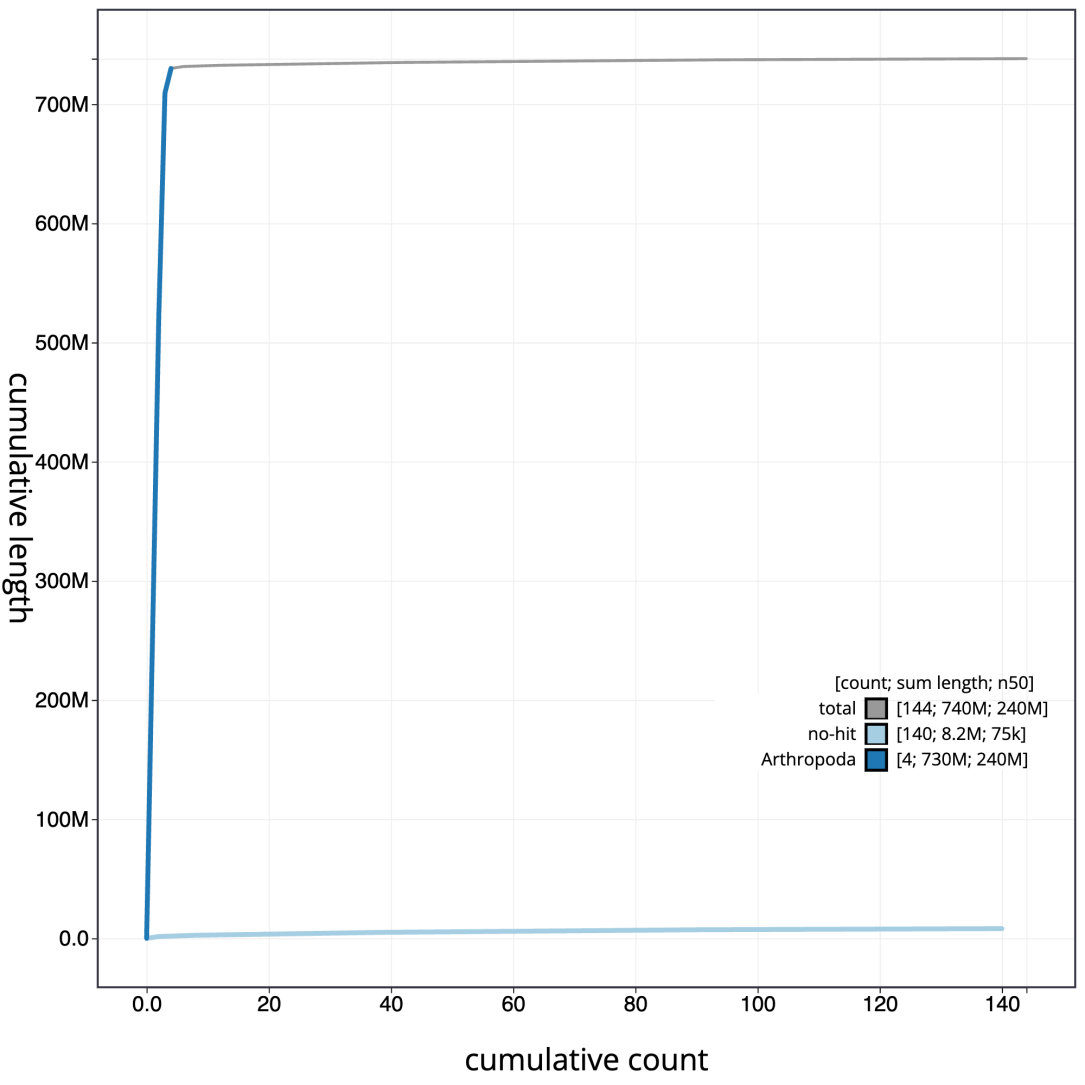
Genome assembly of *Melanostoma scalare*, idMelScal2.1: BlobToolKit cumulative sequence plot. The grey line shows cumulative length for all scaffolds. Coloured lines show cumulative lengths of scaffolds assigned to each phylum using the buscogenes taxrule. An interactive version of this figure is available at https://blobtoolkit.genomehubs.org/view/Melanostoma%20scalare/dataset/CATKHO01/cumulative.

**Figure 5.**
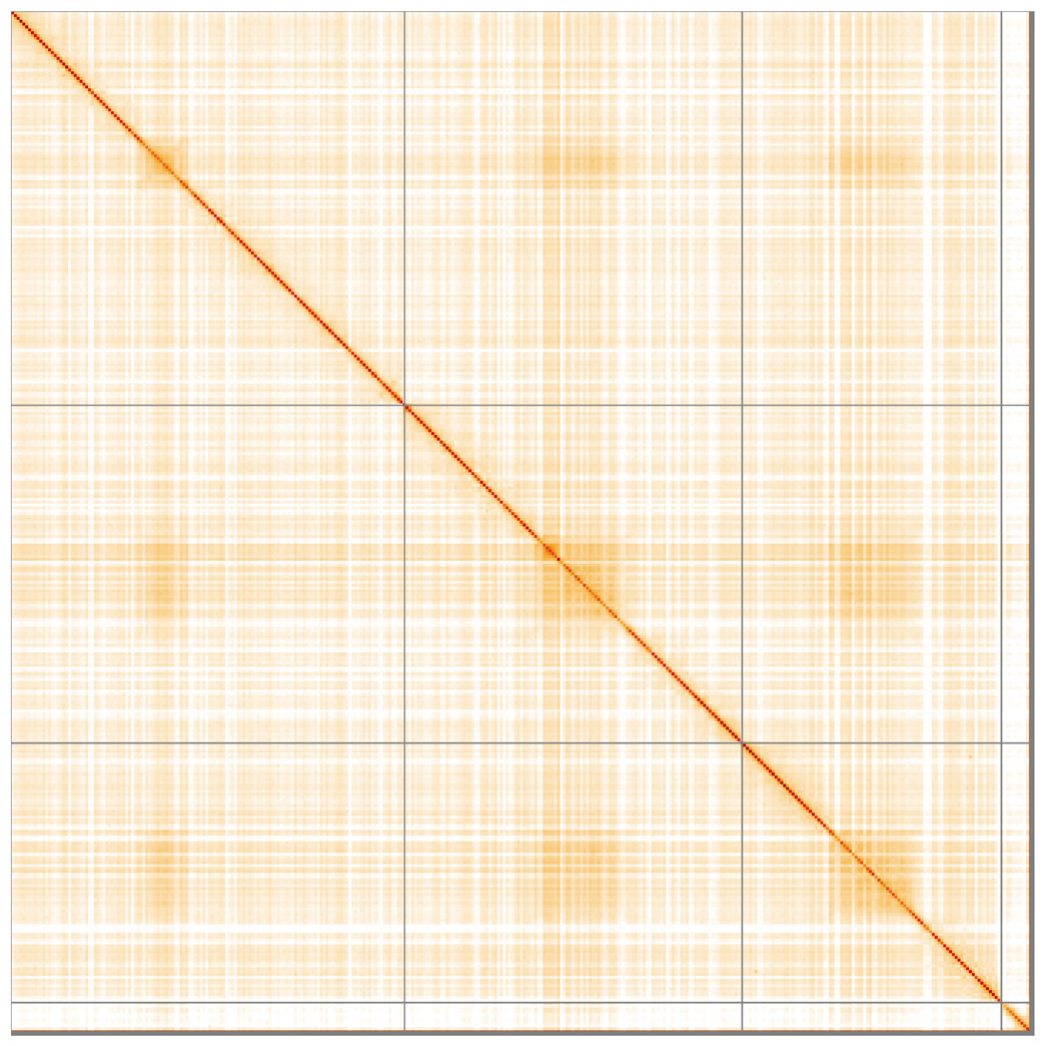
Genome assembly of *Melanostoma scalare*, idMelScal2.1: Hi-C contact map of the idMelScal2.1 assembly, visualised using HiGlass. Chromosomes are shown in order of size from left to right and top to bottom. An interactive version of this figure may be viewed at https://genome-note-higlass.tol.sanger.ac.uk/l/?d=WFlEul_nRvWxkL2eXYGk3A.

The final assembly has a total length of 738.2 Mb in 143 sequence scaffolds with a scaffold N50 of 241.7 Mb ( Table 1). The snailplot in Figure 2 provides a summary of the assembly statistics, while the distribution of assembly scaffolds on GC proportion and coverage is shown in Figure 3. The cumulative assembly plot in Figure 4 shows curves for subsets of scaffolds assigned to different phyla. Most (99.22%) of the assembly sequence was assigned to 5 chromosomal-level scaffolds, representing 3 autosomes and the X and Y sex chromosomes. Sex chromosomes were identified using coverage information. Chromosome Y scaffolds determined by mapping Hi-C data from a female sample to the male assembly. Chromosome-scale scaffolds confirmed by the Hi-C data are named in order of size ( Figure 5; Table 2). While not fully phased, the assembly deposited is of one haplotype. Contigs corresponding to the second haplotype have also been deposited. The mitochondrial genome was also assembled and can be found as a contig within the multifasta file of the genome submission.

**Table 1.** Genome data for *Melanostoma scalare*, idMelScal2.1.

Project accession data		
Assembly identifier	idMelScal2.1	
Species	*Melanostoma scalare*	
Specimen	idMelScal2	
NCBI taxonomy ID	92598	
BioProject	PRJEB53930	
BioSample ID	SAMEA7520053	
Isolate information	idMelScal2, male: head and thorax (DNA sequencing and Hi-C scaffolding) idMelScal1, female: head and thorax (RNA sequencing)	
Assembly metrics *		*Benchmark*
Consensus quality (QV)	60.2	*≥ 50*
*k*-mer completeness	100%	*≥ 95%*
BUSCO **	C:96.4%[S:95.0%,D:1.4%], F:1.0%,M:2.6%,n:3,285	*C ≥ 95%*
Percentage of assembly mapped to chromosomes	99.22%	*≥ 95%*
Sex chromosomes	X and Y chromosomes	*localised* *homologous pairs*
Organelles	Mitochondrial genome assembles	*complete single* *alleles*
Raw data accessions		
PacificBiosciences SEQUEL II	ERR9913038, ERR9913037	
Hi-C Illumina	ERR10659245, ERR9904191	
PolyA RNA-Seq Illumina	ERR9904192	
Genome assembly		
Assembly accession	GCA_949752695.1	
*Accession of alternate* *haplotype*	GCA_949752715.1	
Span (Mb)	738.2	
Number of contigs	701	
Contig N50 length (Mb)	2.3	
Number of scaffolds	143	
Scaffold N50 length (Mb)	241.7	
Longest scaffold (Mb)	281.9	

* Assembly metric benchmarks are adapted from column VGP-2020 of “Table 1: Proposed standards and metrics for defining genome assembly quality” from ( Rhie *et al.*, 2021). ** BUSCO scores based on the diptera_odb10 BUSCO set using v5.3.2. C = complete [S = single copy, D = duplicated], F = fragmented, M = missing, n = number of orthologues in comparison. A full set of BUSCO scores is available at https://blobtoolkit.genomehubs.org/view/Melanostoma%20scalare/dataset/CATKHO01/busco.

**Table 2.** Chromosomal pseudomolecules in the genome assembly of *Melanostoma scalare*, idMelScal2.

INSDC accession	Chromosome	Length (Mb)	GC%
OX456984.1	1	281.87	36.5
OX456985.1	2	241.72	37.0
OX456986.1	3	185.74	37.5
OX456987.1	X	20.67	37.0
OX456988.1	Y	0.95	38.0
OX456989.1	MT	0.02	18.5

The estimated Quality Value (QV) of the final assembly is 60.2 with *k*-mer completeness of 100%, and the assembly has a BUSCO v5.3.2 completeness of 96.4% (single = 95.0%, duplicated = 1.4%), using the diptera_odb10 reference set ( *n* = 3,285).

Metadata for specimens, barcode results, spectra estimates, sequencing runs, contaminants and pre-curation assembly statistics can be found at https://links.tol.sanger.ac.uk/species/92598.

## Methods

### Sample acquisition and nucleic acid extraction

Specimens of *Melanostoma scalare* were netted in Wytham Woods, Oxfordshire (biological vice-county Berkshire), UK (latitude 51.77, longitude –1.34) on 2019-07-31. The specimens were collected and identified by Will Hawkes (University of Exeter) and preserved on dry ice. A male specimen (specimen ID Ox000110, individual idMelScal2) was used for DNA sequencing and Hi-C data and a female specimen was used for RNA sequencing (specimen ID Ox000111, ToLID idMelScal1).

DNA was extracted at the Tree of Life laboratory, Wellcome Sanger Institute (WSI). The idMelScal2 sample was weighed and dissected on dry ice with tissue set aside for Hi-C sequencing. Head and thorax tissue was disrupted using a Nippi Powermasher fitted with a BioMasher pestle. High molecular weight (HMW) DNA was extracted using the Qiagen MagAttract HMW DNA extraction kit. HMW DNA was sheared into an average fragment size of 12–20 kb in a Megaruptor 3 system with speed setting 30. Sheared DNA was purified by solid-phase reversible immobilisation using AMPure PB beads with a 1.8X ratio of beads to sample to remove the shorter fragments and concentrate the DNA sample. The concentration of the sheared and purified DNA was assessed using a Nanodrop spectrophotometer and Qubit Fluorometer and Qubit dsDNA High Sensitivity Assay kit. Fragment size distribution was evaluated by running the sample on the FemtoPulse system.

RNA was extracted from head and thorax tissue of idMelScal1 in the Tree of Life Laboratory at the WSI using TRIzol, according to the manufacturer’s instructions. RNA was then eluted in 50 μl RNAse-free water and its concentration assessed using a Nanodrop spectrophotometer and Qubit Fluorometer using the Qubit RNA Broad-Range (BR) Assay kit. Analysis of the integrity of the RNA was done using Agilent RNA 6000 Pico Kit and Eukaryotic Total RNA assay.

### Sequencing

Pacific Biosciences HiFi circular consensus DNA sequencing libraries were constructed according to the manufacturers’ instructions. Poly(A) RNA-Seq libraries were constructed using the NEB Ultra II RNA Library Prep kit. DNA and RNA sequencing was performed by the Scientific Operations core at the WSI on Pacific Biosciences SEQUEL II (HiFi) and Illumina HiSeq 4000 (RNA-Seq) instruments. Hi-C data were also generated from remaining tissue of idMelScal2 using the Arima2 kit and sequenced on the Illumina NovaSeq 6000 instrument.

### Genome assembly, curation and evaluation

Assembly was carried out with Hifiasm ( Cheng *et al.*, 2021) and haplotypic duplication was identified and removed with purge_dups ( Guan *et al.*, 2020). The assembly was then scaffolded with Hi-C data ( Rao *et al.*, 2014) using YaHS ( Zhou *et al.*, 2023). The assembly was checked for contamination and corrected as described previously ( Howe *et al.*, 2021). Manual curation was performed using HiGlass ( Kerpedjiev *et al.*, 2018) and Pretext ( Harry, 2022). The mitochondrial genome was assembled using MitoHiFi ( Uliano-Silva *et al.*, 2023), which runs MitoFinder ( Allio *et al.*, 2020) or MITOS ( Bernt *et al.*, 2013) and uses these annotations to select the final mitochondrial contig and to ensure the general quality of the sequence.

A Hi-C map for the final assembly was produced using bwa-mem2 ( Vasimuddin *et al.*, 2019) in the Cooler file format ( Abdennur & Mirny, 2020). To assess the assembly metrics, the *k*-mer completeness and QV consensus quality values were calculated in Merqury ( Rhie *et al.*, 2020). This work was done using Nextflow ( Di Tommaso *et al.*, 2017) DSL2 pipelines “sanger-tol/readmapping” ( Surana *et al.*, 2023a) and “sanger-tol/genomenote” ( Surana *et al.*, 2023b). The genome was analysed within the BlobToolKit environment ( Challis *et al.*, 2020) and BUSCO scores ( Manni *et al.*, 2021; Simão *et al.*, 2015) were calculated.

Table 3 contains a list of relevant software tool versions and sources.

**Table 3.** Software tools: versions and sources.

Software tool	Version	Source
BlobToolKit	4.1.7	https://github.com/blobtoolkit/blobtoolkit
BUSCO	5.3.2	https://gitlab.com/ezlab/busco
Hifiasm	0.16.1-r375	https://github.com/chhylp123/hifiasm
HiGlass	1.11.6	https://github.com/higlass/higlass
Merqury	MerquryFK	https://github.com/thegenemyers/MERQURY.FK
MitoHiFi	2	https://github.com/marcelauliano/MitoHiFi
PretextView	0.2	https://github.com/wtsi-hpag/PretextView
purge_dups	1.2.3	https://github.com/dfguan/purge_dups
sanger-tol/ genomenote	v1.0	https://github.com/sanger-tol/genomenote
sanger-tol/ readmapping	1.1.0	https://github.com/sanger-tol/readmapping/tree/1.1.0
YaHS	yahs- 1.1.91eebc2	https://github.com/c-zhou/yahs

### Wellcome Sanger Institute – Legal and Governance

The materials that have contributed to this genome note have been supplied by a Darwin Tree of Life Partner. The submission of materials by a Darwin Tree of Life Partner is subject to the **‘Darwin Tree of Life Project Sampling Code of Practice’**, which can be found in full on the Darwin Tree of Life website here. By agreeing with and signing up to the Sampling Code of Practice, the Darwin Tree of Life Partner agrees they will meet the legal and ethical requirements and standards set out within this document in respect of all samples acquired for, and supplied to, the Darwin Tree of Life Project.

Further, the Wellcome Sanger Institute employs a process whereby due diligence is carried out proportionate to the nature of the materials themselves, and the circumstances under which they have been/are to be collected and provided for use. The purpose of this is to address and mitigate any potential legal and/or ethical implications of receipt and use of the materials as part of the research project, and to ensure that in doing so we align with best practice wherever possible. The overarching areas of consideration are:

•     Ethical review of provenance and sourcing of the material

•     Legality of collection, transfer and use (national and international)

Each transfer of samples is further undertaken according to a Research Collaboration Agreement or Material Transfer Agreement entered into by the Darwin Tree of Life Partner, Genome Research Limited (operating as the Wellcome Sanger Institute), and in some circumstances other Darwin Tree of Life collaborators.

## Data Availability

European Nucleotide Archive: *Melanostoma scalare* (slender grass hoverfly). Accession number PRJEB53930; https://identifiers.org/ena.embl/PRJEB53930. ( Wellcome Sanger Institute, 2023) The genome sequence is released openly for reuse. The *Melanostoma scalare* genome sequencing initiative is part of the Darwin Tree of Life (DToL) project. All raw sequence data and the assembly have been deposited in INSDC databases. The genome will be annotated using available RNA-Seq data and presented through the Ensembl pipeline at the European Bioinformatics Institute. Raw data and assembly accession identifiers are reported in Table 1.

## Author information

Members of the University of Oxford and Wytham Woods Genome Acquisition Lab are listed here: https://doi.org/10.5281/zenodo.4789928.

Members of the Darwin Tree of Life Barcoding collective are listed here: https://doi.org/10.5281/zenodo.4893703.

Members of the Wellcome Sanger Institute Tree of Life programme are listed here: https://doi.org/10.5281/zenodo.4783585.

Members of Wellcome Sanger Institute Scientific Operations: DNA Pipelines collective are listed here: https://doi.org/10.5281/zenodo.4790455.

Members of the Tree of Life Core Informatics collective are listed here: https://doi.org/10.5281/zenodo.5013541.

Members of the Darwin Tree of Life Consortium are listed here: https://doi.org/10.5281/zenodo.4783558.
