# Supplementary material for: The genome sequence of the slender grass hoverfly, Melanostoma scalare (Fabricius, 1794)
Source: Wellcome Open Res. 2023 Oct 23;8:489. [Version 1] doi: 10.12688/wellcomeopenres.20171.1 (PMC11128046; doi:10.12688/wellcomeopenres.20171.1)
Supplement: Peer review history

# Reviewer report: Reviewer response for version 1

**DOI:** 10.21956/wellcomeopenres.22334.r84006

**Published:** 2024-06-19

**Authors:** Kevin M Moran

**Affiliations:** 1 Biology, The University of Memphis, Memphis, Tennessee, USA

The approach presented in this report on the sequencing and assembly of the genome of *Melanostoma scalare *is excellent, and I have no doubt that it will be of great value to researchers.

 It is modern, the raw data is satisfactory, and the methodology is appropriate and understandable. Additionally, the authors have made the raw data and genome publicly available. Based on my own investigation of the data, the resulting genome is high quality and has excellent coverage. 

 It is difficult to determine the identity of the specimen from the provided photograph. While I have no reason to doubt the identification, I urge caution with this group as it can be tricky. There are four species of *Melanostoma* in Northern Europe: *M. scalare*, *M. mellinum*, *M. mellarium* and *M. certum*. *M. scalare *is most easily distinguished from these by longer pruinosity on the arista as compared to the other species.

 It would be helpful if the literary resource used to determine the identity of this species was provided in the text.

Are sufficient details of methods and materials provided to allow replication by others?

Yes

Is the rationale for creating the dataset(s) clearly described?

Yes

Are the datasets clearly presented in a useable and accessible format?

Yes

Are the protocols appropriate and is the work technically sound?

Yes

Reviewer Expertise:

Phylogenetics, Syrphid Taxonomy, Bioinformatics.

I confirm that I have read this submission and believe that I have an appropriate level of expertise to confirm that it is of an acceptable scientific standard.

# Reviewer report: Reviewer response for version 1

**DOI:** 10.21956/wellcomeopenres.22334.r84009

**Published:** 2024-06-18

**Authors:** Claus Vogl

**Affiliations:** 1 Vetmeduni Vienna, Veterinärplatz, Wien, Austria

I am not a fly specialist, but rather a biostatistician/bioinformatician. Reading the note, I was mainly concerned about the identification of the species. However, upon reading further material it seems that both sexes of the species can be identified confidently. It should be made clear in the text that identification of both the male and female sequenced specimens was made with confidence.

 It has turned out (eg in Anopheles mosquitos) by the use of modern genetic methods (such as those employed in the note) that what was once thought to be a single wide-spread species constitutes in fact a species complex. Since both specimens derive from the same location it is unlikely that they actually are different cryptic species (should M scalare be a cryptic species complex). All methods and tools seem state of the art and the description in the text is confident and without fuzz. The DNA data (from the male) are clearly documented with some figures and corresponding text in the results section. On the other hand, the RNAseq data of the female are not documented at all in the results section. I wonder if this intentional.

 Small stuff:

 "Background" section: take out "as" from "... perhaps as because ..."

Are sufficient details of methods and materials provided to allow replication by others?

Yes

Is the rationale for creating the dataset(s) clearly described?

Yes

Are the datasets clearly presented in a useable and accessible format?

Yes

Are the protocols appropriate and is the work technically sound?

Yes

Reviewer Expertise:

biostatistics, bioinformatics, theoretical population genetics

I confirm that I have read this submission and believe that I have an appropriate level of expertise to confirm that it is of an acceptable scientific standard.

# Reviewer report: Reviewer response for version 1

**DOI:** 10.21956/wellcomeopenres.22334.r81955

**Published:** 2024-05-25

**Authors:** Andrés López Rubio

**Affiliations:** 1 Tecnológico de Antioquia – University Institute, Antioquia, Colombia

The article shows the publishing for the complete genome of *Melanostooma scalare *with 99.2 % coverage.

 The methods on the paper are well described.  It would be good to show the results on RNA sequencing. Any replicate was deposited in a Biological collection?

 I found this paper useful since sequencing and produce genomic data from non model species is having great impact in the understanding of the biology of every species

Are sufficient details of methods and materials provided to allow replication by others?

Yes

Is the rationale for creating the dataset(s) clearly described?

Yes

Are the datasets clearly presented in a useable and accessible format?

Yes

Are the protocols appropriate and is the work technically sound?

Yes

Reviewer Expertise:

Phylogeny, Entomology, Evolutionary biology

I confirm that I have read this submission and believe that I have an appropriate level of expertise to confirm that it is of an acceptable scientific standard.

# Reviewer report: Reviewer response for version 1

**DOI:** 10.21956/wellcomeopenres.22334.r84012

**Published:** 2024-05-22

**Authors:** Darren J Obbard

**Affiliations:** 1 The University of Edinburgh, Edinburgh, Scotland, UK

This data note reports the sequencing and assembly of the genome of *Melanostoma scalare* for the “Darwin Tree of Life” programme. In common with other data notes from this research effort, the reporting is standardised and quite brief. As such, I have very few comments to make.

 The approach is state-of-the-art, the raw data appear to be of a suitably high quality, and the assembly methods are appropriate. The public availability of raw data and genome assembly are appropriate. The resulting genome is likely to be of very high quality, and I have no doubt that it will be of great value to any researchers working on the comparative or evolutionary genomics of insects.

 I have a couple of very minor suggestions

- In addition to the photograph of the sequenced male specimen, it would be very nice to have images of both the male and female in life.
- The introduction is well written, and not excessively brief. However, it would be nice to have a little more detail on the distribution, ecology, or behaviour (if papers exist)
- How does the sequenced mtDNA compare to the previously published one? Are they the same length with similar gene content?
- The assembly of X and Y is great – but both appear to be very small. Is that expected? Or, is it more likely that one or both are mostly unassembled due to repeats? It would be great to have a comparison with (say) expected genome size based on DNA content, and/or previously published cytology if any exists

Are sufficient details of methods and materials provided to allow replication by others?

Yes

Is the rationale for creating the dataset(s) clearly described?

Yes

Are the datasets clearly presented in a useable and accessible format?

Yes

Are the protocols appropriate and is the work technically sound?

Yes

Reviewer Expertise:

Evolutionary genomics of Diptera and their pathogens.

I confirm that I have read this submission and believe that I have an appropriate level of expertise to confirm that it is of an acceptable scientific standard.
